# Applied behavioral analysis for the skill performance of children with autism spectrum disorder

**DOI:** 10.3389/fpsyt.2023.1093252

**Published:** 2023-04-26

**Authors:** Alan Patricio da Silva, Italla Maria Pinheiro Bezerra, Thaiany Pedrozo Campos Antunes, Matheus Paiva Emidio Cavalcanti, Luiz Carlos de Abreu

**Affiliations:** ^1^Laboratory of Study Design and Scientific Writing, FMABC University Center, Santo André, SP, Brazil; ^2^Postgraduate Department, Master's Program in Public Policy and Local Development, Santa Casa de Misericórdia de Vitória-EMESCAM, Vitória, ES, Brazil; ^3^Department of Nutrition, Federal University of Espírito Santo-UFES, Vitória, ES, Brazil

**Keywords:** autism, autism spectrum disorder, applied behavior analysis, behavior, cognition

## Abstract

**Introduction:**

Autism spectrum disorder (ASD) has characteristics that have been observed to develop over time, such as the difficulty of affective, sensory, and emotional processing, which trigger some problems during childhood, limiting children's development. Applied behavior analysis (ABA) is among the therapeutic approaches for ASD, in which treatment can be tailored according to the patient's objectives.

**Objective:**

Based on ABA, we aimed to analyze the therapeutic strategy for independence in different skill performance tasks of patients diagnosed with ASD.

**Method:**

This is a retrospective observational case series study including 16 children diagnosed with ASD who received ABA-based treatment at a therapeutic clinic in Santo Andre city, São Paulo State, Brazil. Individual task performance of different skill domains was registered in the ABA+ affective intelligence^®^ software throughout the 12 months (from January 2021 to January 2022) of routine treatment.

**Results:**

The evolution of skills was observed between the T0 and T1 intervals, with improved skills over the observed period.

**Conclusion:**

The strategy based on the ABA methodology improved children's skill performance over the observed period.

## Introduction

Autism spectrum disorder (ASD) is a neurological condition characterized by deficits in communication and social interaction, specific interests and repetitive actions. Furthermore, they present difficulty of affective, sensory, and emotional processing, which triggers a series of complications for the individual and might affect child development (1).

An ASD diagnosis is based on differences observed in behavior between children with ASD and other children, with changes in development and skill acquisition, mainly to establish social relationships and interactions (2, 3). Although currently there are no subcategories, the 10th International Classification of Diseases (ICD-10) established the spectrum (F-84). Its diagnostic criteria stipulate that ASD can be identified at around 3 years of age; however, some signs are already identifiable before this age (2–4).

Among the characteristics most marked in the behavioral pattern, the difficulty in communicating is essential to recognize the presence of ASD. Inflexibility in adapting to routine changes and changing ritualized behavioral patterns are prominent aspects of ASD (5–7). There is a significant impairment in verbal and non-verbal communication. The absence of eye contact, social isolation, and a tendency toward stillness during interactions and activities such as play are also commonly observed. In socialization environments, an individual with ADS does not play or interact with others, tending toward isolation. This behavior damages socialization because it happens daily (5–7).

There is still no consensus on the etiology of ASD. It is believed that many genes and factors, including environmental factors, contribute to the construction of this disorder (3, 8). Regarding behavior, clinical manifestations are diverse and are influenced by sensory and environmental factors. To obtain a viable path for the therapy of autism, it is necessary to understand and identify the factors that affect the individual and his/her pattern of response to stimuli (8). Therefore, various approaches can favor the individual's performance in activities of daily living and social relationships.

Applied behavior analysis (ABA) is a therapeutic approach for treating ASD that can be tailored according to the patient's condition. It was first applied to people with ASD by Lovaas (9). This method explains behaviors and plans strategies to increase, decrease, create, eliminate, or improve behaviors (10). The strategy based on the ABA methodology was aimed at carrying out interventions during behavioral treatment. Skills are taught through the presentation of an instruction or a tip, with the child's help through a hierarchy of help (11–13). ABA was developed with the aim for the patients to consistently succeed in their task attempts, either with help or independently. Children with significant difficulties can learn and overcome challenges to become independent and integrated into the community.

Behavior is the relationship between the subject, his/her responses, and the environment, including the antecedent and consequent stimuli to the behavior presented on a given occasion. The analysis starts from the concepts of the approach: behavior, antecedent or discriminative stimulus, response, and consequence or reinforcement (10, 14, 15).

If a child does not achieve the expected level of performance during the learning process, the professional reviews the teaching methodology and modifies it for the best execution and development that the individual can achieve to improve the quality of the skills acquired and this gradually enhances his/her social skills, games, academic activities, and self-care (10, 16).

The training record gives the therapist an overview of the patient's development throughout treatment. It allows for a systematic evaluation of the applied therapeutic strategy, describing the skills manifested throughout the intervention period and identifying the clinical evolution of the skills and their post-treatment clinical outcomes (16). Thus, this study aimed to analyze the therapeutic strategy based on ABA on independence for the performance of tasks in different skill sets of patients diagnosed with an autism spectrum disorder.

## Methods

The research ethics committee of the Centro Universitário FMABC approved this study under registration number 1,833,631. It is a retrospective observational study of a case series including children diagnosed with autism (ICD-10: F-84) and undergoing treatment at a therapeutic clinic in Santo André, a metropolitan region of São Paulo state, Brazil. This clinic is attended by 50 patients with ASD, of which 16 were selected for convenience to compose this study sample.

Regularly treating children diagnosed with ASD in this multi-professional therapeutic clinic is based on ABA. The current study participants underwent treatment at least three times per week between January 2021 and January 2022. The data were collected in January 2022. Authorization was requested for data usage, and to prioritize ethical criteria and preserve participants' identities, they were identified by “participant number” (example: Participant 1).

The data of the different skill domains during treatment were registered with the software ABA+ intelligence affective^®^, which works as a digital diary. ABA+ intelligence affective^®^ facilitates integration and ongoing supervision of therapies used daily by the multi-professional team (16). The software focuses on usability, optimizes therapy routines, allows better recording of the results, and uses ABA to monitor the patient. It provides a record of the skills that comprise the individual's development. Each member of the therapeutic team can fill in this “diary” with his/her own goals and steps to reach the final purpose, reassessing and modifying it to meet the specific demands of each patient.

Each skill domain has its own set of intervention elements that enable the determination of specific learning and development targets. Thus, it is possible to measure the performance in domains and observe the individual's singularity in the execution of the tasks, verifying evolution in their clinical condition and acquisition of competence in each observed domain. The formal analysis weighed children's performance in the respective domains and considered the patient's execution curve in the observed period.

The following variables of the macro groups were analyzed: ADL skills, social skills, and academic skills available in the ABA+ software, and their sub-variables corresponding to the respective domains, which, as an example, are listed in Table 1.

**Table 1:** Structure of some domains available in the ABA+ software.

**Macro domain—skill**	**Tasks**
Social	Play functional
	Accept peer behavior change request
	Play using games with rules
ADL	Wash hands
	Wash the dishes
	Use of the bathroom
Academic	Identify colors
	Use the pencil
	Write your name

The macro domains have several tasks; therefore, different tasks were collected for the children, respecting the acquired skills.

The skill domains evaluated in the initial and final phases of the clinical intervention consist of task training that stimulates the development of determinant attributes. They are as follows.

### Attention skills

Waiting, making eye contact, maintaining shared attention, sitting, visual tracking, and exploring textures.

### Imitation skills

Imitating actions with objects, motor actions, making simple discrimination, phono-articulatory movements, gross movements while standing, motor movements spontaneously, fine motor movements, gross motor movements, and gross motor movements of two steps while standing, among others.

### Receptive language skills

Putting objects in a container, listening behavior by feature, listener behavior by class, listener behavior by function, determining own preferences, identifying figures, and identifying figures by function, among others.

### Expressive language skills

Pointing toward desired items, increasing verbal requests, increasing spontaneous vocal requests, completing an object's function, completing words that describe everyday activities, verbally completing a sentence, communicating by picture exchange, phonological awareness, giving instruction or an explanation of how to do something, and describing actions.

### Academic skills

Hand-eye coordination, drawing shapes, pairing figures, pairing objects, pairing objects with figures, writing their names, identifying colors, naming numbers, and naming vowels, among others.

### Motor skills

Skating, walking on a line, blowing a whistle, riding a bike/scooter, playing and using motor movements, walking, kicking/throwing, coloring, cutting, and pasting, among other available workouts.

### Activity of daily living (ADL) skills

Opening the fridge, opening a snap button, opening a belt buckle, opening the cupboard to get kitchen items, opening velcro, tying shoelaces, making the bed, showering, brushing teeth, taking off pants, shorts, pantyhose, skirt, underwear, or panties, standing and sitting, washing hands, washing dishes, and using the bathroom, among others.

### Academic skills

Relating numbers and quantities, solving fractions, solving math problems, answering “open mind” challenges, sequencing numbers, serializing, adding, subtracting, a unit of time, completing a sequence, completing sets, completing words, counting, copying, copying syllabic families, copying phrases, copying numerals from 1 to 10, and copying simple words, among others.

### Social skills

Accepting a request to change the behavior, waiting for the turn to speak, helping, helping peers, taking the bus, walking on the sidewalk and not on the street, answering the bell, crossing the street, knocking on the door before entering, playing autonomously with toys that require several different motor actions, playing creatively, playing follow the master, functional play, functional and social play with various objects with other children spontaneously, playing symbolically about various life events using verbal scripts, using games with rules, seeking physical interaction, calling people by name, and behaving appropriately in different social situations, among other training available on the bench.

### Group skills

Waiting for the turn in activities, raising a hand to get the teacher's attention, organizing own material, standing in line, following daily routines, working independently on non-academic skills, and cooperating in pairs/trios/teams.

### Nurturing sensory skills

Trying new foods.

As an analysis parameter, we used the individualized teaching and treatment program (17); this instrument gathers information from the individual's initial assessment parameter and analyzes the individual's acquired competencies, pointing out the skills that should be explored. The participants were analyzed according to their performance throughout therapy and the variables observed in the ADL, attention, academic, and social skills groups. The children's profiles and the performance curves for the tasks during the study period were identified.

The evaluation of overall development was examined by the Psychoeducational Profile–Revised (PEP-R) instrument to verify behaviors and abilities through information related to the functioning in the areas of imitation, perception, gross motor, eye-hand integration, verbal cognitive, and cognitive performance. It also identifies possible behavioral modifications in relationship and affection, playing and interest in materials, sensory responses, and language (17, 18).

The profile obtained in the assessed fields provides valuable information regarding the nature of the child's learning abilities and difficulties. It also offers a third result identified as “in the process of being acquired,” in which the patient indicates some knowledge of what is needed to complete the task but does not have the concrete skill to perform it successfully.

## Results

Participants were between 3.8 and 10.8 years old, and 75% of them were boys (10). Table 2 shows the distribution of cases regarding age group and sex.

**Table 2:** Frequency distribution by age group and sex.

**Age group**	**Amount of children**	**Sex**	
		**Boys**	**Girls**
3–5	1	1	0
5–7	8	5	3
7–9	2	2	0
9–11	5	2	3

For this study, we presented only the group of skills the participants most frequently trained during the observed period.

Tables 3–5 present the distribution of frequencies of task attempts made and successes reached for each skill observed in the children, done with or without help. “Maximum attempts” represents the number of opportunities offered to the participant to accomplish a particular task, and “accomplished attempts” represents the actual number of tasks accomplished by the participant, either with help or independently. In these tables, all children could independently perform at least two of the proposed tasks in more than half of their successful attempts. Academic skills were the ones that the children were most able to perform independently (Table 3), followed by social skills (Table 4) and ADLs (Table 5). Tasks that children were able to perform independently are highlighted in bold.

**Table 3:** Participants' task training results for academic skills.

**Participant**	**Task**	**Skill: academic**			
		**Attempts**		**Success**	
		**Maximum**	**Accomplished**	**With help**	**Independent**
1	Pair objects	3,225	2,627	51.50%	48.50%
2	Completing sets	435	228	21.93%	**78.07%**
	Identifying concepts	237	221	4.98%	**95.02%**
3	Writing numerals	337	337	62.91%	37.09%
	Doing homework	6	6	16.67%	**83.33%**
	Reading sentences	142	124	21.77%	**78.23%**
	Writing simple words	134	130	16.15%	**83.85%**
4	Matching/grouping images and objects	1,761	357	80.95%	19.05%
	Matching/grouping objects by color	1,301	280	40%	60%
5	Aggrouping	104	80	21.25%	**78.75%**
	Reading sentences	160	151	59.60%	40.40%
	Reading simple words	788	536	5.78%	**94.22%**
6	Copying vowels	348	348	57.76%	42.24%
	Copying	1,061	647	12.67%	**87.33%**
	Writing simple words	277	199	11.06%	**88.94%**
7	Identifying predecessor and successor	1,415	224	32.14%	67.86%
	Telling	1,210	586	6.14%	**93.86%**
	Identifying numbers	237	152	14.47%	**85.53%**
8	Identifying vowel clusters	193	50	10%	90%
	Identifying concepts	942	457	49.45%	50.55%
	Telling	1,236	746	37.40%	62.60%
9	Pairing words/pictures	120	72	4.17%	**95.83%**
	Completing the sentence	2	110	19.09%	**80.91%**
	Identify concepts	1,125	619	26.17%	**73.83%**
	Telling	298	293	18.43%	**81.57%**
10	Understanding the relationship between quantities and symbols	180	141	43.97%	56.03%
	Matching letters in the name itself	450	77	10.39%	**89.61%**
11	Identifying numbers	421	376	29.79%	**70.21%**
	Reading vowel encounters	76	76	38.16%	61.84%
	Aggrouping	30	30	10%	**90%**
	Adding	572	322	26.09%	**73.91%**
12	Cutting and pasting	785	540	84.07%	15.93%
13	Completing words	400	82	52.44%	47.56%
	Copying simple words	288	69	7.25%	**92.75%**
	Doing homework	923	213	60.09%	39.91%
14	Completing sets	45	42	9.52%	**90.48%**
	Copying simple words	242	191	0%	**100%**
	Writing sentences	70	67	23.88%	**76.12%**
15	Copying simple words	90	26	0%	**100%**
	Categorizing	213	73	0%	**100%**
	Identifying sequences	259	198	5.05%	**94.95%**
	Aggrouping	165	95	3.16%	**96.84%**
16	Identifying numbers	803	226	42.48%	57.52%
	Telling	600	178	81.46%	18.54%
	Completing a sequences	692	185	90.27%	9.73%

Tasks that children were able to perform independently are highlighted in bold.

**Table 4:** Participant's task training results for ADL skills.

**Participant**	**Task**	**Skill: ADL**			
		**Attempts**		**Success**	
		**Maximum**	**Accomplished**	**With help**	**Independent**
1	Hair care	42	42	90.48%	9.52%
	Hand washing	21	21	61.90%	38.10%
	Showering	105	105	79.05%	20.95
	Using cutlery during meals	130	130	70%	30%
2	Dishwashing	924	919	69.97%	30.03%
	Hand washing	283	278	55.76%	44.24%
	Bathroom use	216	216	46.30%	53.70%
4	Participating in all steps of handwashing	10	8	25%	**75%**
	Nose care	39	39	41.03%	58.97%
	Hair care	270	265	60.75%	39.25%
5	Bathroom use	896	883	6.34%	**93.66%**
	Teeth brushing	2,150	1,808	12.89%	**87.11%**
	Hand washing	318	300	11%	**89%**
6	Copying vowels	348	348	57.76%	42.24%
	Copying	1,061	647	12.67%	**87.33%**
	Writing simple words	277	199	11.06%	**88.94%**
7	Wearing long pants, shorts, tights, and skirt	98	92	59.78%	40.22%
	Bathroom use	159	156	18.59%	**81.41%**
	Hand washing	461	448	73.66%	26.34%
8	Hand washing	134	134	63.43%	36.57%
	Wearing shoes	212	212	70.75%	29.25%
	Taking shoes off	45	45	15.56%	**84.44%**
	Teeth brushing	404	404	90.10%	9.90%
9	Wearing shoes	56	56	19.64%	**80.36%**
	Teeth brushing	620	614	58.14%	41.86%
10	Bathroom use	300	299	62.21%	37.79%
	Teeth brushing	224	224	41.96%	58.04%
	Hand washing	316	316	60.44%	39.56%
11	Trying new foods	97	97	71.13%	28.87%
	Dishwashing	495	485	5.36%	**94.64%**
	Teeth brushing	208	207	16.43%	**83.57%**
12	Teeth brushing	933	869	11.05%	**88.95%**
	Showering	55	55	94.55%	5.45%
	Choosing the right clothing	120	120	20%	**80%**
13	Showering	212	209	48.80%	51.20%
	Taking off a long-sleeved or short-sleeved shirt or tank top	1,005	842	2.26%	**97.74%**
	Hand washing	738	607	2.80%	**97.20%**
	Recognizing the correct side of clothing	184	184	17.39%	**82.61%**
14	Hand washing	323	276	8.70%	**91.30%**
	Bathroom use	512	358	17.60%	**82.40%**
	Dishwashing	430	430	2.79%	**97.21%**
	Teeth brushing	2,025	1,621	3.64%	**96.36%**
15	Choosing food	29	29	0%	**100%**
	Bathroom use	950	936	17.41%	**82.59%**
	Dishwashing	532	526	26.81%	**73.19%**
	Teeth brushing	1,575	1,523	12.54%	**87.46%**
	Setting the table before the meal	145	132	0.76%	**99.24%**
16	Teeth brushing	184	184	78.80%	21.20%
	Taking off a long-sleeved or short-sleeved shirt or tank top	150	149	51.01%	48.99%

Tasks that children were able to perform independently are highlighted in bold.

**Table 5:** Participants' task training results for social skills.

**Participant**	**Task**	**Skill: social**			
		**Attempts**		**Success**	
		**Maximum**	**Accomplished**	**With help**	**Independent**
1	Increasing vocal requests	1,540	798	31.45%	**68.55%**
2	Perform role play	43	31	0%	**100%**
	Following rules and instructions in group situations	140	105	90.48%	9.52%
	Playing follow the master	80	75	53.33%	46.67%
	Changing shift	77	67	40.30%	59.70%
3	Spontaneously practicing civility	808	749	38.99%	61.01%
	Identifying emotions	252	161	14.29%	**85.71%**
	Giving and accepting compliments	136	118	40.68%	59.32%
	Negotiating conflicting interests	347	296	60.81%	39.19%
4	Giving an object to the partner when they ask for it	2,088	442	87.33%	12.67%
	Receptively identifying emotions (happy, sad, angry)	25	24	100%	0%
	Maintaining engagement in sensory and social routines for 2	1,474	271	54.98%	45.02%
5	Responding to greeting with gestures or vocalization.	35	30	63.33%	36.67%
	Answering questions	462	302	4.30%	**95.70%**
	Asking peers for behavior change	73	30	30%	**70%**
	Spontaneously practicing civility	175	116	17.24%	**82.76%**
6	Playing functional	548	251	38.65%	61.35%
7	Spontaneously practicing civility	2,303	310	75.81%	24.19%
	Answering social questions	763	412	38.83%	61.17%
8	Calling by name	134	115	99.13%	0.87%
	Playing functional	258	105	47.62%	52.38%
	Identifying emotions	671	336	14.58%	**85.42%**
9	Playing functional	232	111	66.67%	33.33%
	Greeting	50	18	16.67%	**83.33%**
	Spontaneously practicing civility	486	161	88.20%	11.80%
10	Use courtesy terms: “please,” “thank you,” and “sorry.”	1,104	260	20.77%	**79.23%**
	Expressively identifying affection from photographs	8	8	12.50%	**87.50%**
	Giving an object to the partner when they ask for it	832	153	8.50%	**91.50%**
11	Identifying emotions	348	329	14.89%	**85.11%**
	Spontaneously practicing civility	255	219	23.74%	**76.26%**
	Answering miscellaneous questions	1,061	549	31.88%	68.12%
	Recognizing and naming own and others' emotions	100	88	67.05%	32.95%
	Waiting for the turn in activities	107	58	3.45%	**96.55%**
	Cooperating in pairs/trios/teams	14	14	7.14%	**92.86%**
12	Waiting turn in activities	174	73	27.40%	**72.60%**
	Spontaneously practicing civility	1,031	364	44.51%	55.49%
	Calming down	867	233	59.66%	40.34%
	Agreeing or disagreeing with opinions	195	82	20.73%	79.27%
13	Accepting the request for peer behavior change	192	116	28.45%	**71.55%**
	Cooperating in pairs/trios/teams	30	11	18.18%	**81.82%**
	Playing functional	44	20	0%	**100%**
14	Accepting requests for peer behavior change	156	110	51.82%	48.18%
	Expressing positive and negative emotions	50	48	16.67%	**83.33%**
	Answering questions	100	94	41.49%	58.51%
	Waiting turn in activities	265	183	34.43%	65.57%
15	Agreeing or disagreeing with opinions	16	16	0%	**100%**
	Asking questions	160	160	20%	**80%**
	Accepting a request for peer behavior change	16	16	0%	**100%**

Tasks that children were able to perform independently are highlighted in bold.

Attention skills also appeared frequently, but only three children had performed them: Participants 1, 10, and 16. The tasks that required attention skills were *waiting, making eye contact, following one-step instructions, following instructions to “stop” or “wait” unassisted, identifying body parts, imitating large movements while standing, and imitating fine motor movements*.

Participant 10, in the “*following instructions to ‘stop' or ‘wait”'* task, from 90 accomplished attempts, completed 16.67% attempts with support and 83.33% independently. Participant 16, in the “*imitating fine motor movements*” task, from 92 accomplished attempts, completed 2.17% attempts with support and 97.83% independently. As for attention skills, in the “*following one-step instructions*” task, from 3,020 attempts, 66.49% attempts were executed with support and 33.51% independently. Participant 1 was the most dependent on help for most of the tasks. Participant 1 was the most dependent on help for most of the tasks. His best performance was in carrying out activities related to ADL and academic skills.

## Discussion

In this study, the outcomes from a therapeutic process using ABA to treat individuals diagnosed with ASD were analyzed. Specific training data for each child's skills were presented.

Each child is unique, and the skill training starting point is unique. Therefore, it is not possible to establish parametric relationships for the group of individuals, considering that each individual diagnosed with autism manifests characteristics that hinder the homogeneity of strategies as well as the same procedures for such different individuals, even if they belong to the same age group.

These findings allow us to evaluate the performance of individuals undergoing treatment in a therapeutic clinic, analyzing individual performance in each skill and aspect of their biopsychosocial development. The results make it possible to define specific strategies for each skill, identify where there is a more significant competence deficit, and explore new solutions for better development. There is a concern among caregivers for the acquisition of skills by their children throughout their development (19), with support strategies and tools that improve the acquisition of skills for practical life and training in systematic training skills for children with ASD to achieve adequate development. Proposals shared between professionals and caregivers can constantly encourage the child to acquire skills (20).

In the general analysis of the cases, it was observed that all children could independently complete at least two of the proposed tasks in more than half of their successes. Academic skills were the ones that children were most able to perform independently, followed by social skills and ADLs.

Academic learning improves cognitive development and memory and expands the child's intellectual repertoire (21, 22), which favors their learning process and interpersonal relationships.

With caregivers' support as intermediary process agents, daily living activities in a therapeutic setting enrich learning and generalization to other spaces (20). It benefits the progress of self-care skills and interactions in the family environment where the child is included in adolescence and adulthood (10, 23).

When a child can independently exercise social and participation skills in the therapeutic setting, those behaviors are reproduced in other relationships in the child's living settings, with better permanence in social participation spaces (15, 17). Social skills training helps the child break down attitudinal and behavioral barriers, which can provide experiences that improve the quality of life and social relations (17), favoring learning of new aptitudes and constructing bonds and interpersonal relationships. Souza et al. (24) state that ABA improves the social and affective skills of children with ASD because it reduces repetitive behavior and stereotypes by strengthening socially accepted modes and modifying non-accepted ones.

Studies involving the ABA strategy compared to other stimulation protocols demonstrate that this methodology is efficient in several aspects, as it positively influences a modification in the child's behavior, which favors its development (25). The application of therapeutic strategies supported by the ABA can obtain promising outcomes in developing individuals with ASD, considering the experience provided and the control of the skill training environment that favors skill acquisition (19, 26–29).

Different protocols can encourage clinical practices to improve the cognitive skills of people with different health conditions, helping to develop strategies to improve the quality of life of people with disabilities (30). An example was observed in the Therapeutic Effects of Exercise in Adults with Amnestic Mild Cognitive Impairment study, which demonstrated the processes for developing and validating best practices for promoting the quality of life through improvements in cognition, memory, orientation, and executive functions (30, 31).

Skills training and its aiding tools seek to improve and expand their response to stimuli and to improve the child's engagement in activities that involve interaction (27, 28). It has been observed that music therapy and protocols associated with the ABA methodology influence the results achieved by children with autism in school and family environments.

The use of technology can favor interactive processes when strategies are supported by relatives for the application of behavioral interventions, especially in the conditions of the actual situation. A family engaged in the behavioral intervention and the mediation of specialist professionals create teaching conditions to obtain a correct answer from their child, which expands the chance of more right answers from their children, allowing for more opportunities for learning (20).

It is essential to highlight the historical moment in which this study was carried out. The disruption of everyday life with the impact of the COVID-19 pandemic shows a potential psychological impact on children with neurodevelopmental disorders and their caregivers. Thus, children who maintained regularity in treatment benefited from the performance of their skills, which could reduce the effects of the social isolation period (32–35).

Individuals living with ASD have symptoms that differentiate them from the contemporary social model, which reduces their quality of life (26). The results showed that the therapeutic strategy combined with the systematic data recording by the ABA + software allowed the evaluation of the performance of children with ASD undergoing ABA treatment. This strategy allowed for these children's development and independence during tasks involving different skill groups.

Thus, it is up to each health professional in the multidisciplinary team to define the best practices based on the analysis of everyone's development to help in acquiring the necessary skills for social participation and practical life, reflecting on the individual's quality of life with ASD.

Future studies may strengthen the findings for the development of strategies to meet the specific needs in the performance of skills of individuals with ASD in their various aspects, such as ADL, social skills, attention, and academic skills, through a systematic training approach focused on the acquisition of these skills.

## Conclusion

The therapeutic strategy using applied behavioral analysis allowed children with an autism spectrum disorder to independently perform most of the proposed tasks, especially those related to academic, social, and activities of daily living skills.

## Data availability statement

The raw data supporting the conclusions of this article will be made available by the authors, without undue reservation.

## Ethics statement

The studies involving human participants were reviewed and approved by Centro Universitário FMABC Parecer CEP 1,833,631. Written informed consent to participate in this study was provided by the participants' legal guardian/next of kin. Written informed consent was obtained from the individual(s), and minor(s)' legal guardian/next of kin, for the publication of any potentially identifiable images or data included in this article.

## Author contributions

AS and LA designed the study, coordinated the study, and recruited the participants. MC analyzed the study data and drafted the manuscript. TA helped with study design, data analysis, and wrote the final version of the manuscript. AS, TA, and IB edited the manuscript. All authors contributed to the article and approved its submitted version.
